# Investigating the effect of clinical history before electrocardiogram interpretation on the visual behavior and interpretation accuracy of clinicians

**DOI:** 10.1038/s41598-019-47830-0

**Published:** 2019-08-05

**Authors:** Alan Davies, Simon Harper, Markel Vigo, Caroline Jay

**Affiliations:** School of Computer Science, University of Manchester, Manchester, UK

**Keywords:** Cardiology, Computer science

## Abstract

We examine the impact of the presentation of a patient’s clinical history on subsequent visual appraisal and interpretation accuracy of electrocardiograms (ECGs). Healthcare-practitioners (N = 31) skilled in 12-lead ECG interpretation took part in a repeated-measures experiment with counterbalancing viewing 9 ECGs on a computer screen in two separate conditions: with/without an associated patient-history. A Hellinger-distance calculation was applied using a permutation test to eye-movement transitions at two granularity levels: between the ECG leads, and between smaller grid-cells, whose size was determined via data-driven clustering of the fixation points. Findings indicate that presentation of clinical-history does affect accuracy of interpretation in one ECG. Visual-behavior differed as a function of both history presentation and accuracy when considering transitions between the data-driven grid units (using a fine granularity, and able to show attention to parts of the waveform). Differences in visual-behavior at waveform level demonstrate an influence of patient-history and expertise that are not detected at the lead level. Visual-behaviour differs according to whether a patient-history is presented, and whether a clinician provides an accurate interpretation. This difference is evident in how the waveform itself is viewed, and is less present at the coarse granularity of visual transitions between leads. To understand how clinicians interpret ECGs, and potentially other medical images, visual transitions should be considered at a fine level of granularity, determined in a data-driven fashion.

## Introduction

The electrocardiogram (ECG) is a common test used in many areas of clinical practice, with over 300 million ECGs carried out per year in Europe^1^. Failure to correctly interpret an ECG can lead to an incorrect medical diagnosis and subsequent administration of inappropriate (or no) treatment^1,2^. The ECG displays waves of electrical activity resulting from the depolarization and repolarization of cells in the myocardium^3^. The 12-lead ECG represents this signal data in 12 different channels, called ‘leads’. Automated/computerized methods that are used to generate a clinical interpretation have been shown to be less accurate than humans^4^, despite ongoing improvements in automated interpretation since their inception in the 1960’s^5^. As a result of this, many cardiology organizations, such as the American College of Cardiology and the American Heart Association counsel against the use of computer interpretation of ECGs without expert human oversight^6^. An incorrect computerized interpretation is more likely to be accepted by over-readers than primary readers. This may result from not having direct access to the patient, or other relevant clinical information, such as the patient’s clinical history^7^. Clinical mismanagement, including potentially dangerous or inappropriate treatment has occurred when less experienced practitioners fail to identify interpretation errors and accept automated diagnostic information without question^4^.

Current research in the medical domain is considering approaches to enhance human accuracy, rather than supersede it with technology. Such methods keep the human ‘in-the-loop’, and promise to enhance human and machine interaction by leveraging the advantages of both paradigms. Observation of visual behaviour via eye tracking, which provides an objective means of assessing perception, cognition and performance, is a useful tool in linking human and machine interpretation. Methods include machine learning analysis combining human gaze information with image content to enhance breast imaging diagnostics^8^.

Eye-tracking has previously been applied to medical images (such as x-rays and mammograms) to understand how they are viewed by both experts and novices^9–11^. Eye-tracking has also more recently been used to gain insights into differences between the visual behavior of experts and novices, and those making correct and incorrect interpretations as they view ECGs^12–14^.

An eye-tracking study by Wood *et al*.^13^ also explored the effect of including clinical history with some of the ECGs as they were viewed by experts (consultant emergency medics, n = 10) and novices (final year medical students, n = 10). Sixteen ECGs were used with clinical histories provided for half (n = 8) of the ECGs. No time limit was imposed during the study. Areas of Interest were defined around leads that 2 clinicians believed to be the most important leads for interpreting the condition presented for each ECG. Findings suggest that the clinical history had no significant effect on ECG abnormality detection. The study did not however present each ECG with and without the associated clinical history, so the results may have been affected by confounding factors, such as learning effect/fatigue which are acknowledged by the authors.

In this paper we explore whether presenting clinical history affects interpretation accuracy and visual behavior, addressing some of the limitations of the study carried out by Wood *et al*.^13^. We analyse visual transition behaviour at a coarse level of granularity (between leads) and at a finer level of granularity, between units of a grid whose size is determined in a data-driven fashion, via clustering of the gaze data. The results show that transitions between units of the grid vary significantly according to both history presentation and interpretation accuracy, but no difference is detected when considering visual transitions between leads. This indicates that the differences in visual behaviour occur in how people view the morphology of the waveform, rather than how they make comparisons between leads. Although we do not see a difference in interpretation accuracy according to history presentation, the fact that significant differences in fine-grained visual behaviour are detected indicates that presenting history does make a difference to how people interpret ECGs, and that to understand its effects via observation of visual behaviour, it is important to use a method that captures within-lead transitions, as well as between-lead transitions.

## Objective

The aim of the study was to examine the effect of clinical history on subsequent ECG interpretation accuracy and visual transition behavior. The ECG stimulus was segmented into areas of interest (AOIs) for performing the transition analysis in two ways. An AOI was mapped onto each ECG lead in a top-down fashion; this is contrasted with a data-driven, bottom-up segmentation method that uses clustering to determine the size of grid cells that serve as AOIs. We explore how these different approaches affect our ability to understand the effects of history and accuracy on visual behavior.

## Materials and Methods

### Study design

A within-subjects (repeated measures) experimental design was used. Participants (N = 31) were shown nine different 12-lead ECGs (Table 1) in arbitrary sequence in two conditions: with a preceding brief history of the presenting complaint (Table 2); or alone with no history. The sequence of the presentation of the ECGs was not randomized, in order to keep the history associated with the correct subsequent ECG. Counterbalancing was used to determine which way the stimuli were presented (i.e. with history first or last). Participants wrote their interpretation on an answer sheet. No time limit was imposed, allowing participants as much time as required to make an interpretation. The type of clinical case history and level of detail we provide is similar in nature to other training materials and level of information available in real life clinical scenarios. In training materials, on-line examples and books etc. a brief patient history is often added to accompany the presented ECG. An example of this can be seen in^15^.

**Table 1:** ECGs used in experiment.

• Anterolateral ST-segment elevation MI
• Left bundle branch block
• Lateral ST-segment elevation MI
• Atrial fibrillation
• Right bundle branch block
• Inferior ST-segment MI with atrial fibrillation
• Anterior ST-segment elevation MI
• High lateral ST-segment elevation MI
• Inferolateral ST-segment elevation MI

**Table 2:** ECG and associated clinical history (history of presenting complaint.

**Anterolateral STEMI:** 31 year old male. Heavy cocaine use. 30 seconds of severe chest pain. Pain free at present
**LBBB:** 65 year old male. Smoker. Sweaty. Vomiting. Discomfort in jaw and shoulders
**Lateral STEMI:** 67 year old female. Previous PCI (Percutaneous Coronary Intervention) several years previous. Central chest pain approximately 1 hour in duration
**Atrial fibrillation:** 78 year old male. Palpitations on and off for 2 days. Presented with syncope
**RBBB:** 35 year old female. Recent long haul flight. Sharp stabbing chest pain and shortness of breath
**Inferior STEMI and AF:** 80 year old male. Resus. Return of spontaneous circulation after x2 DC cardioversion
**Anterior STEMI:** 40 year old male with sudden pressure to chest and very clammy. 30 minutes from symptom onset
**High lateral STEMI:** 47 year old male. Acute central chest pain worsening in waiting room. Radiating to right shoulder
**Inferolateral STEMI:** 91 year old female with shortness of breath. Close to collapse. Sweating and mild chest discomfort

*“ECG 87: A 30-year-old man, who had had brief episodes of palpitations for at least 10 years, was seen during an attack in the A&E department and this is his ECG. What is the rhythm, and what would you do immediately, and in the long term?”* (Hampton, 2003, p173).

### Participants

**Figure 1:**
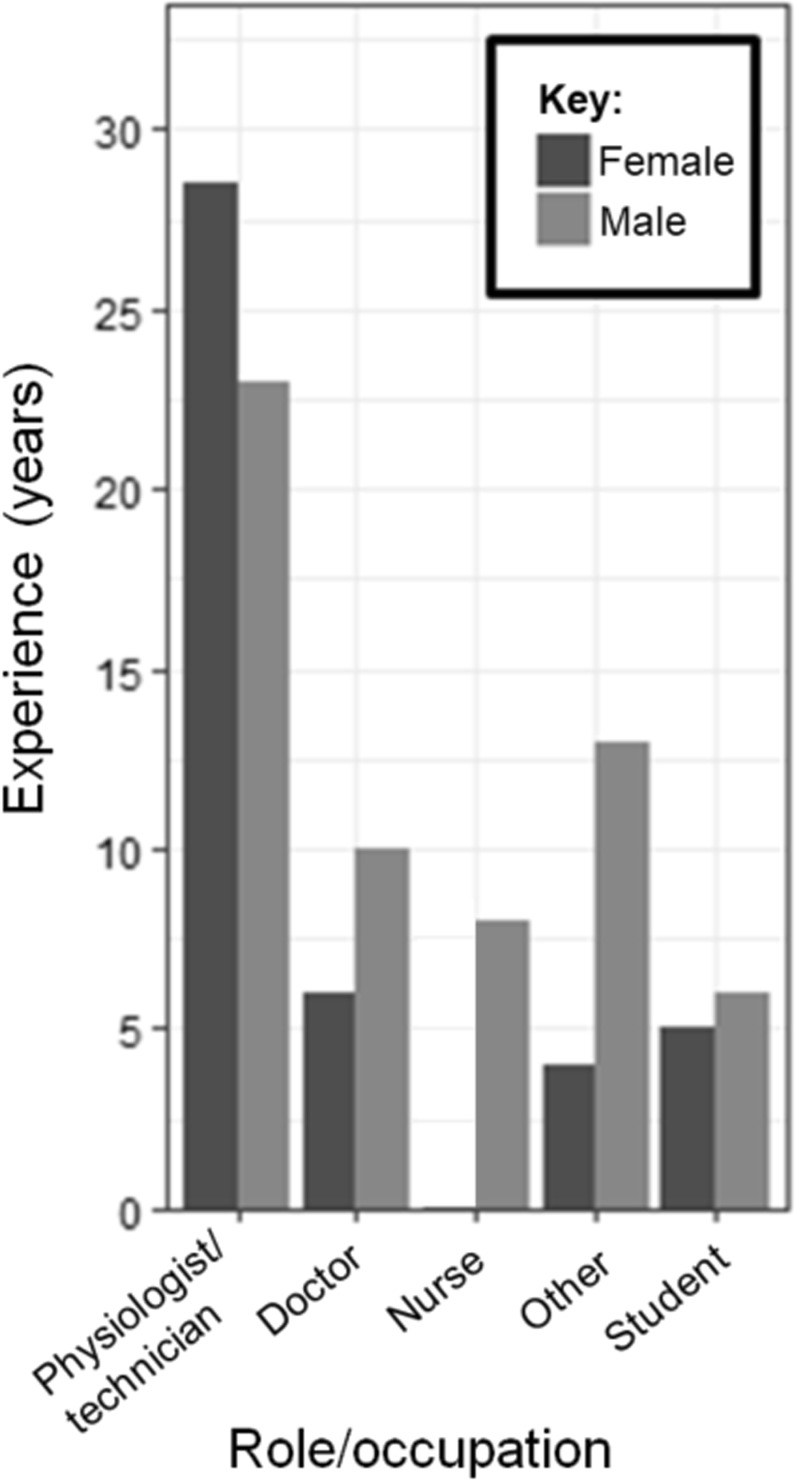
Participants’ roles and years of experience.

Thirty one participants (males = 13, females = 18, *Mdn* age = 28, *SD* = 8.1) were recruited from hospitals and universities in the North West and West of England by word of mouth, using “snowball” sampling. All of the participant’s self-identified as having received some training in ECG interpretation, and/or carried out ECG interpretation as part of their clinical role. Informed consent was obtained from all participants, and ethical approval was obtained from the University of Manchester Research Ethics Committee (CS65e). All methods were performed in accordance with the relevant guidelines and regulations. Five main categories of role were defined as: physiologists/technicians (n = 16), doctors (n = 2), nurses (n = 2), students (n = 7) and other (n = 4). Figure 1 summarizes these categories, along with their experience and sex.

The main forms of training in ECG interpretation received by participants included lectures/seminars, workshops and being taught on the job by colleagues. 9.7% of participants (n = 3) received just 1–5 hours of training, juxtaposed to this the same number of participants (n = 3) had over 100 hours of training. The rest of the participants were somewhere between: n = 6 (19%), [6–10 hours]; n = 1 (3%), [11–20 hours]; n = 5 (16%), [21–30 hours], n = 12 (39%) [>30 hours].

### Stimuli

The ECGs were selected from anonymized patients (Table 1), predominantly suffering from myocardial infarctions (heart attacks). The ECGs were provided by a local hospital A&E department, following verification of diagnosis. A summary of the associated medical histories concerning the patients’ presenting complaint was also provided anonymously. A number of other conditions were added as discriminators, in order to provide enough variety to prevent participants from assuming all the stimuli were representative of myocardial infarctions (MIs). The MIs were chosen as they require visual examination of specific leads, or combinations of leads in order to make a correct interpretation, thusly providing some ground truth from medical training literature as to where participants should look in order to make a correct interpretation.

### Method

A Tobii X2-60 eye-tracker and Tobii studio software version 3.2.0 were used with the I-VT fixation filter (default settings) to capture participants gaze data. Participants sat comfortably in a quiet room at a distance of ≈60 cm as per manufacturer’s recommendations. Using a within-subject design, each participant viewed all 9 ECGs twice, once with the associated clinical history and once without. Counterbalancing was used to alter the presentation sequence to show either the ECGs with history first or last. This was done to reduce the potential impact of confounding factors, such as learning effect and fatigue on participants.

**Figure 2:**
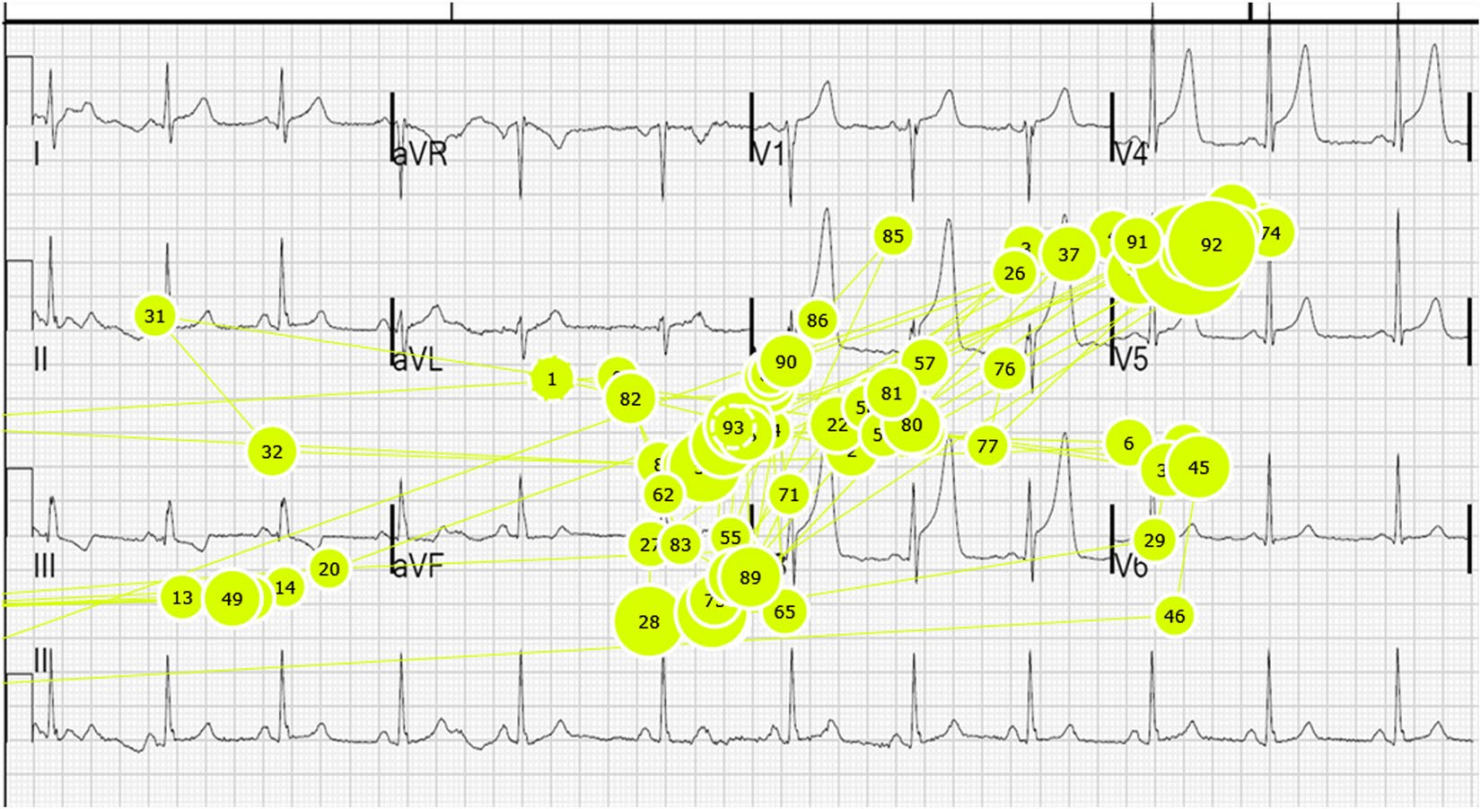
A representative scanpath from a single participant (16F) showing attention focused around the anterior leads of an ECG displaying features of an anterior STEMI.

Eye-tracking metrics (fixation duration and count) across the whole of each ECG were compared between the two groups (history and no-history), along with the overall and per-group accuracy of interpretation. These metrics are used as proxies for attention (fixation count) and increased cognitive load (fixation duration). Accuracy of interpretation was determined by comparing the answers given with the ground-truth of the condition, and was scored as being either correct or incorrect. In order to be ‘correct’ an answer of clinical quality was required, for example the condition Atrial Fibrillation (AF) would need to be (AF, or atrial fibrillation) not arrhythmia or SVT. Common variations of conditions and acronyms were also accepted as correct (i.e. left bundle branch or LBBB). Two visual transition analyses were carried out: one at the level of the leads, where AOIs were applied by the researcher; the other using a data-driven bottom-up approach to determine the size of cells for a grid with the grid cells serving as AOIs. Figure 2 shows a ‘gaze plot’ from a single participant. The participants’ scanpath can be seen represented in sequence by number, with the circle indicating a fixation. The larger the circle, the greater the fixation duration.

For assessing lead transitions, AOIs were created using Tobii studio software and mapped onto each of the ECG leads by the researcher. The bottom up approach applied the Density-based spatial clustering of applications with noise (DBSCAN) algorithm^16^ to cluster visual fixations. A grid was applied to the stimulus, the dimensions of whose cells were derived from the optimal diameter obtained from the DBSCAN algorithm^17^.

For both methods of stimulus segmentation (lead and grid), the frequency of visual transitions was determined (within and between the leads or grid cells) for the two conditions (saw history first and did not see history first). The two transition matrices representing the two conditions were converted into Markov chains, and similarity was determined by calculating the Hellinger distance between them (Equation 1) as a measure of difference between the probability distributions. Additional comparison groups were generated by shuffling participants from the two groups of interest into additional equally sized groups 10,000 times with a permutation test in order to generate enough sampling data to compare against the initial result. A distribution was created of the Hellinger distance from each sub-group comparison, which was then compared with the distance between the initial groups of interest^17^.

The same procedure was used to determine whether there was a difference in visual behaviour as a function of accuracy. As accuracy could not be determined in advance, the groups were created on a post hoc basis for each stimulus in each condition.

$$H(P,Q)=\frac{1}{\sqrt{2}}\sqrt{\sum _{i=1}^{k}\,{(\sqrt{{p}_{i}}-\sqrt{{q}_{i}})}^{2}}$$

**Equation 1**: Hellinger distance for discrete probability distributions.

## Results

We report the results of the participants’ overall accuracy as a function of history presentation, followed by the fixation duration and count metrics. Finally, we present the results of the visual transition permutation tests. Where multiple statistical tests were carried out, the generally more conservative Bonferroni correction was used to control for type I error rate^18^. All analysis presented was carried out using the R project for statistical computing version 3.1.1^19^.

### Accuracy

**Figure 3:**
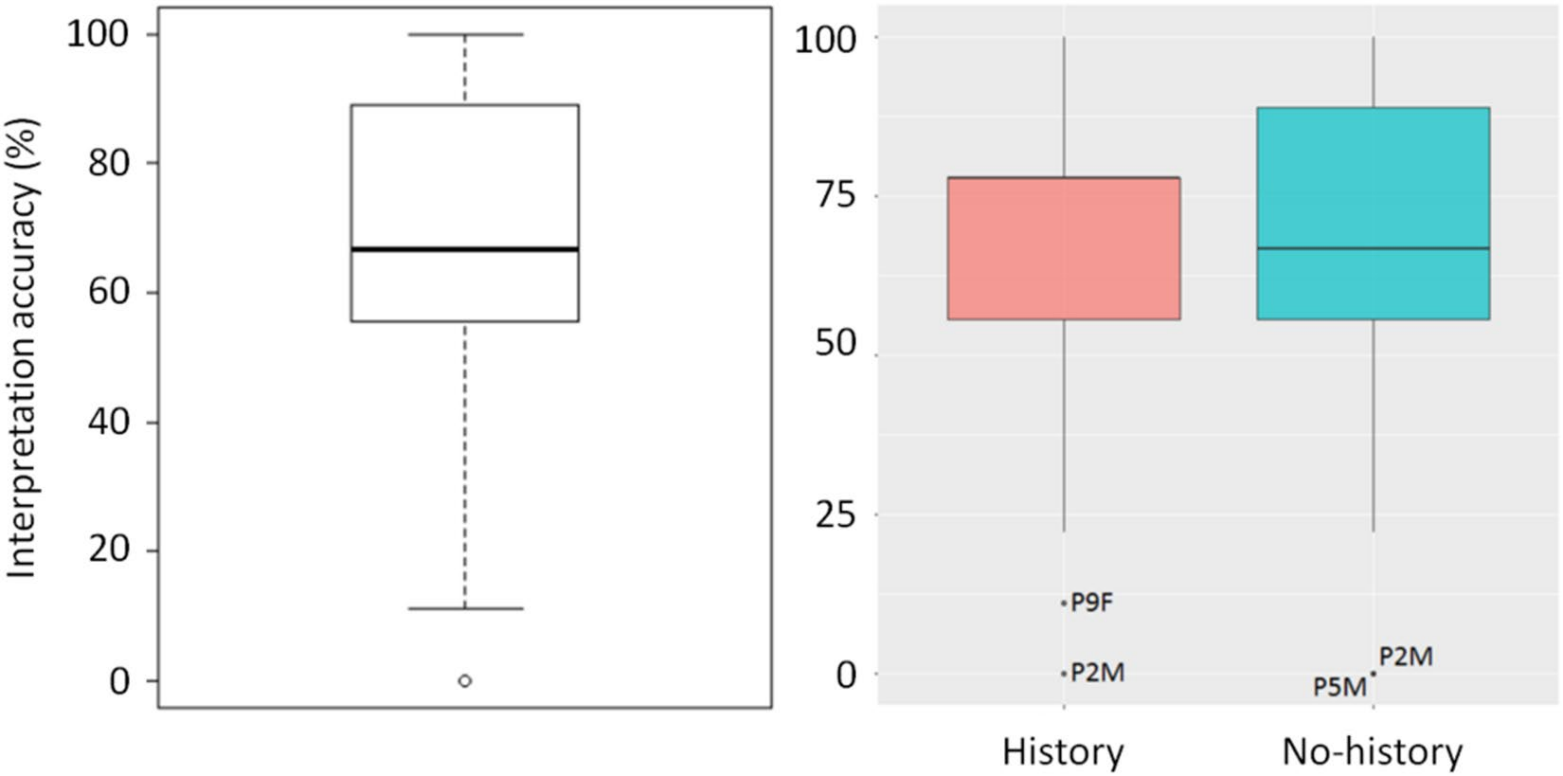
(left) Boxplot of overall percentage accuracy (all participants), (right) accuracy per group (outliers labeled).

The average interpretation accuracy across all ECGs (Figure. 3 left) for all participants was 64% (*SD* = 27). Figure 3 (right) shows the overall accuracy for the two groups (history and no-history). Participant 2 (P2M) failed to correctly interpret any of the ECGs in either condition (Participant 2 - the only non-clinical participant did not have a clinical role in ECG interpretation but identified that they did interpret ECGs as part of their role as a medical scientist). The proportion of correct and incorrect results (Wilcoxon test) was not significantly different between the history and no-history groups (*V* = 84.5, *p* = 0.720).

When examining the results on a per-ECG basis using a McNemar’s chi-squared for repeated measures data (Table 3), a difference between the history and no-history group can be seen in the LBBB stimuli. The history for this ECG - *“65 year old male. Smoker. Sweaty. Vomiting. Discomfort in jaw and shoulders”*, describes a potential acute coronary syndrome. And LBBB in the presence of chest pain should be treated as a medical emergency^3^.

**Table 3:** Results of McNemar’s chi-squared test per ECG on accuracy of interpretation between history and no-history groups.

ECG	χ^2^	p-value
Anterolateral STEMI	6.533	0.010
LBBB	8.533	0.003*
Lateral STEMI	0.037	0.847
AF	7	0.008
RBBB	3.125	0.077
Inferior STEMI + AF	0.862	0.353
Anterior STEMI	0.571	0.449
High lateral STEMI	4.8	0.449
Inferolateral STEMI	5.121	0.023

*Note: DF* = 1, *p < 0.005 (Bonferroni correction).

### Fixation duration

**Figure 4:**
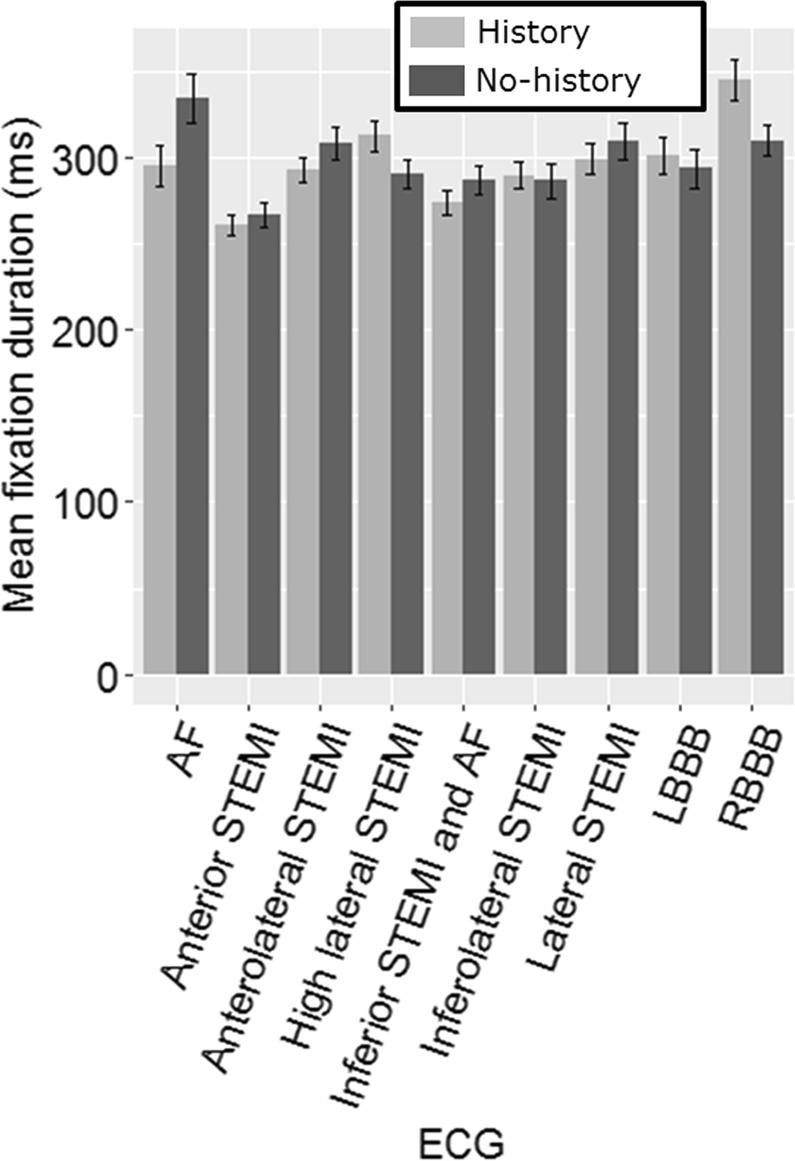
Mean fixation duration for both conditions per ECG (error bars = SE).

Mean fixation duration – the average length of a single fixation – can be used as a proxy for cognitive load, where longer fixations indicate increased cognitive load^20^. The mean fixation duration did not differ significantly between the two groups when compared with a Wilcoxon test with Bonferroni correction (α = 0.005) (Figure. 4 and Table 4), suggesting that the presence of clinical history had no significant impact on the cognitive load entailed in interpreting the ECG.

**Table 4:** Results of Wilcoxon tests, comparing fixation count and duration between the history and no-history groups.

ECG	Fixation Count		Fixation duration	
	*V*	*p-value*	*V*	*p-value*
LBBB	230.5	0.786	241	0.871
Lateral STEMI	219.5	0.974	194	0.440
AF	230.5	0.786	183	0.318
RBBB	292.5	0.220	274	0.404
Inferior STEMI + AF	323	0.063	307	0.129
Anterior STEMI	280	0.179	231	0.983
High lateral STEMI	311.5	0.043	303	0.151
Inferolateral STEMI	275	0.387	247	0.776
Anterolateral STEMI	274	0.398	234	0.983

*Note:* Bonferroni correction α = 0.005.

### Fixation count

**Figure 5:**
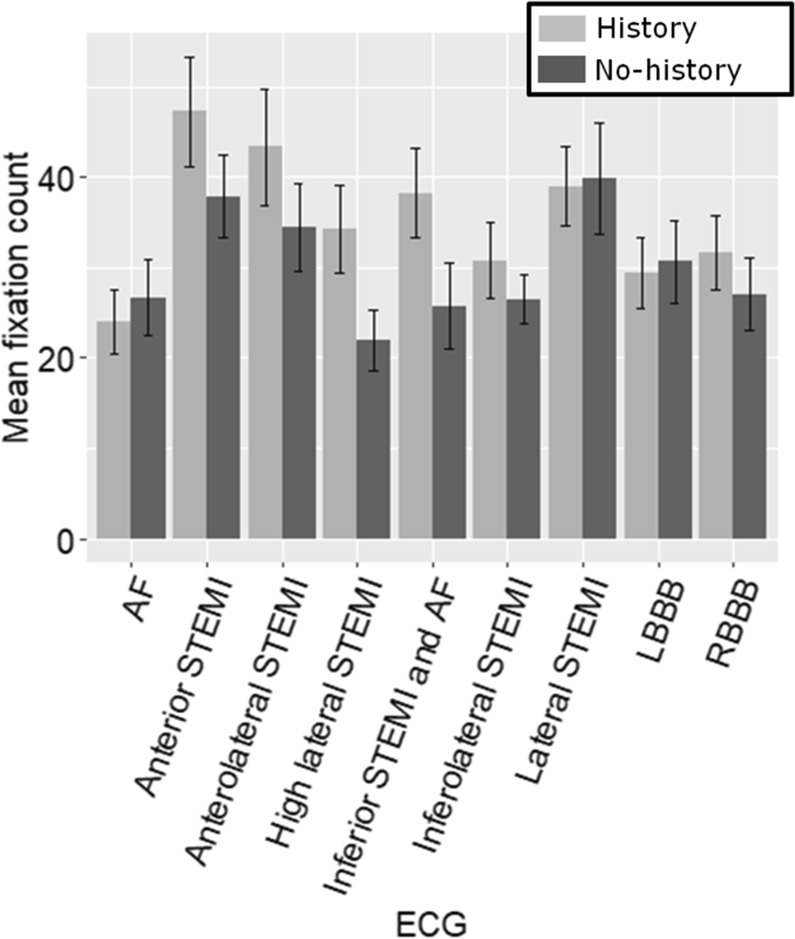
Mean fixation count for both conditions per ECG (error bars = SE).

A Wilcoxon test with Bonferroni correction (α = 0.005) was also carried out on the frequency of fixations (fixation count) for the history and no-history conditions. No significant differences were found between the conditions in any of the ECGs (Figure. 5 and Table 4).

### Permutation tests

In order to determine if the differences in transitional behavior represent real differences between the groups, permutation tests were used to see if there was something “special” about the initial group differences when compared to the differences in groups generated at random with a permutation test. When comparing visual transitions in the history and no history conditions (Table 5), we see that significant differences are detected when using the grid cells as AOIs, but not the leads. The largest noticeable differences were seen in the subgroup analysis that compared accuracy sub-groups between the primary history and no-history groups. This was the case for:

- correct and incorrect
- correct and correct
- incorrect and incorrect

**Table 5:** Results of Hellinger Distance calculation permutation test (10,000 permutations) for the lead and grid cell AOIs per ECG (history/no-history).

ECG	Lead AOI				Grid cell AOI			
	*M (SD)*	*d*	*Hd*	*p-value*	*M (SD)*	*d*	*Hd*	*p-value*
Anterolateral STEMI	0.45 (0.02)	0.1	0.12	0.578	0.8 (0.01)	4.1	0.83	<0.001
Inferolateral STEMI	0.5 (0.02)	0.2	0.13	0.860	0.8 (0.01)	8.6	0.90	<0.001
High lateral STEMI	0.5 (0.02)	0.1	0.14	0.702	0.5 (0.01)	23.4	0.68	<0.001
Anterior STEMI	0.4 (0.02)	1.0	0.12	0.557	0.8 (0.11)	13.5	0.91	<0.001
Inferior STEMI/AF	0.4 (0.02)	0.4	0.13	0.729	0.8 (0.01)	10.2	0.94	<0.001
RBBB	0.5 (0.02)	0.5	0.16	0.188	0.8 (0.01)	9.0	0.89	<0.001
Atrial fibrillation	0.5 (0.02)	0.01	0.16	0.811	0.5 (0.01)	24.1	0.66	<0.001
Lateral STEMI	0.4 (0.03)	0.8	0.13	0.627	0.8 (0.01)	10.7	0.89	<0.001
LBBB	0.5 (0.03)	1.6	0.16	0.553	0.8 (0.01)	7.1	0.86	<0.001

*Note: Hd* = Hellinger distance, *AOI* = Area Of Interest.

between the group that saw history first and the group that saw history last (Appendix).

**Figure 6:**
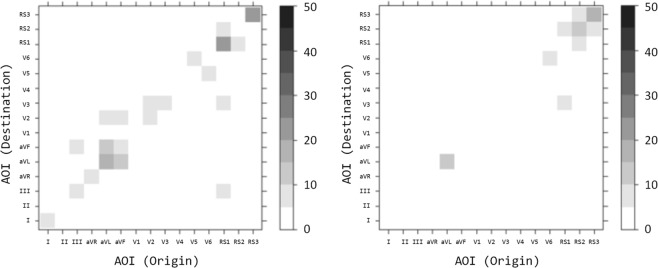
Lead transition matrices for the RBBB condition. (Left) saw history then ECG, (right) saw ECG alone (normalized by max value).

When looking at the lead transitions, the lowest p value (0.2) can be seen for the Right Bundle Branch (RBBB) condition as shown in Figure. 6. Further to history and no-history a sub-group analysis was carried out comparing accuracy sub groups to the primary history and no-history groups (see appendix). Differences can be seen between the accuracy groups in most conditions. One condition, RBBB in the sub-group analysis (Appendix) shows differences in accuracy in both the top-down (*Hd* = 0.67, *p* = 0.06) and bottom-up groups (*Hd* = 0.81, *p* < 0.001). This stimulus also had a clinical history especially evocative of a pulmonary embolism (PE). When considering the differences based on accuracy of interpretation alone (Table 6), greater differences (*Hd*) and smaller p-values can be seen between correct and incorrect participants using the grid method. A significant difference (*p* < 0.05) can be seen in the Anterior ST-segment Elevation Myocardial Infarction (STEMI) suggesting that regardless of history there was a greater difference in visual transitional behaviour between the correct and incorrect groups for this condition.

**Table 6:** Results of Hellinger Distance calculation permutation test (10,000 permutations) based on accuracy of interpretation per ECG.

ECG	Lead AOI				Grid cell AOI				Group sizes	
	*M (SD)*	*d*	*Hd*	*p-value*	*M (SD)*	*d*	*Hd*	*p-value*	1 *(n)*	2 *(n)*
Anterolateral STEMI	0.5 (0.05)	0.03	0.52	0.444	0.8 (0.02)	0.7	0.78	0.261	22	6
Inferolateral STEMI	0.5 (0.03)	0.5	0.55	0.286	0.8 (0.01)	0.8	0.81	0.216	21	7
High lateral STEMI	0.5 (0.03)	1.0	0.48	0.836	0.5 (0.01)	0.3	0.49	0.347	9	19
**Anterior STEMI**	**0.4** (**0.02)**	**0.5**	**0.39**	**0.286**	**0.8** (**0.01)**	**2.4**	**0.79**	0.010*	**16**	**12**
Inferior STEMI/AF	0.4 (0.02)	1.7	0.40	0.965	0.9 (0.01)	1.4	0.87	0.070	15	12
RBBB	0.5 (0.03)	0.9	0.47	0.812	0.8 (0.01)	0.004	0.80	0.502	18	10
Atrial fibrillation	0.5 (0.03)	0.5	0.52	0.654	0.5 (0.01)	1.5	0.46	0.938	21	7
Lateral STEMI	0.4 (0.03)	1.6	0.37	0.955	0.8 (0.01)	2.0	0.77	0.973	14	14
LBBB	0.5 (0.03)	0.3	0.54	0.360	0.8 (0.01)	0.9	0.79	0.183	20	7

*NOTE:* *p < 0.05, *Hd* = Hellinger distance, *AOI* = Area Of Interest.

## Discussion

We set out to examine the effect of clinical history on both accuracy of interpretation and visual behavior. Did the way people viewed the ECG subsequently change because of the inclusion or exclusion of a patient’s clinical history? Further to this we explored the impact of using AOIs at two levels of granularity. Researcher-defined AOIs were mapped onto the ECG leads. These leads represent different separate semantic areas that contain views of the heart’s electrical activity based on the direction of the electrical impulses in relation to the position of the surface electrodes^3,21^. We contrasted this with a bottom-up data-driven method for segregation of the stimulus space into different AOIs. In all cases the grid cell dimensions were smaller than the lead level AOIs. Certain ECG leads can be of more or less importance in making a correct interpretation depending on the underlying condition. ECG literature and training texts regularly cite different leads as being the best locations to view morphological changes associated with specific pathology^3,21^. To this end the STEMI (ST-elevation myocardial infarction) conditions are of particular relevance, as cross referencing changes to the ST-segment of the ECG waveform in certain lead territories is necessary to distinguish a STEMI from other differential diagnosis involving ST-elevation, such as pericarditis^21^. Knowledge from ECG training material and clinical practice also highlights the need to potentially cross-reference leads, but additionally also cross-reference individual components of the ECG waveform within a lead itself. This requires the study and comparison of different waveform components, such as the various waves, intervals and segments. Which part of the waveform that pathological morphological changes occur in depends on the underlying condition itself^3^. Without making an arbitrary decision about which sub-components of the waveform to focus on, it becomes practically infeasible to map all parts of potential interest for subsequent analysis. To overcome arbitrary selection, we introduced a grid method to segregate the stimulus space based on values from a clustering algorithm.

This suggests that for these stimuli - details within the leads were more significant in terms of identifying visual behavior differences between the history and no-history groups. Clustering has been used to segregate stimuli in previous work^22,23^. Previous approaches have however created AOIs that differ in size and/or overlap making direct comparison of the regions difficult or inappropriate.

Medical history provides useful information about the underlying medical condition. Previous work has identified that an increased level of rhythm assessment can be accounted for by having prior knowledge of the patient^7^, making this a useful factor to consider when examining interpretation accuracy. The effect of clinical history on accuracy is still debatable with some studies determining an effect, i.e. Hatala *et al*.^24^, who found a 4–12% improvement in accuracy with clinical history and Wood *et al*.^13^ who found no difference. Some of the variation in these findings could relate to the strength of association between know symptoms and certain conditions. An example can be seen in the sub group analysis of the RBBB condition in the study presented in this paper with a clinical history that is very suggestive of a pulmonary embolism secondary to a deep vein thrombosis (DVT). Without this history a RBBB pattern is all that can be discerned from the ECG alone. Of the 11 times PE was stated or queried as a possible interpretation, only one participant referred to a possible PE in the no-history group, with the other 10 participants all being in the group that saw the history. This suggests that at least in this case the clinical history was highly suggestive of a specific pathology. This could also be a sign of confirmation bias, with practitioners seeking confirmatory information to back up their initial diagnosis^25^.

The commonly used eye-tracking metrics (such as fixation duration/count) that are used as proxies of behavior^20^ did not detect any significant differences between the conditions in terms of the average length of time they were fixated on or the number of fixations made.

The systematic application of history to all conditions, and presenting them both with and without the history has overcome some of the limitations of the study carried out by Wood *et al*.^13^. The grid method of AOI generation allows for examination of visual behavior at a finer level of granularity than the lead-based analysis due to the increased number of AOIs generated. There may be several grid cells inside a single lead, making the analysis sensitive to within-lead transitions in this case. It is possible that the grid generated could have been larger than the lead. In this case it could be entire regions, or combinations of leads that would be captured.

The clinical history does appear to have an impact on ECG interpretation accuracy in some cases and on visual transitions between leads. One reason why this effect is not more widespread, despite the stimuli being primarily representative of myocardial infarctions that would require lead comparisons is that the bottom-up saliency of the STEMI conditions overrides the vaguer top-down knowledge provided by the histories describing chest pain (Table 2). In contrast to this, interpretation accuracy and visual behavior are both affected at the level of the waveform morphology. This indicated that at the level of the waveform participants react differently on a cognitive and perceptual level. This also makes some sense from a clinical perspective, as a history of chest pain may prompt visual analysis of the ST-segment within leads.

The implications of this are that visual analysis of the waveform components is influenced by the clinical history provided and does impact the way the waveform is subsequently perceived. This implies that regardless of the need to cross reference leads the cross-referencing of the waveform components themselves is of more importance for accuracy of interpretation.

From a clinical standpoint, practitioners are trained to look for morphological changes in the waveform itself, and so intra-lead transitions that are not detected when analyzing transitions at the level of the lead are more relevant and discriminatory of accuracy. This suggests that the level of granularity that is best for analyzing differences in visual behavior is one that considers behavior within leads, as well as between them, which is supported by clinical training courses and texts that teach practitioners to look at the components of the waveform and compare them.

## Limitations

There were several limitations present in the study. By not restricting the time for each task, in order not to rush people and make them behave in an atypical way to how they would normally when interpreting an ECG, we are unable to account for any variance in accuracy due to differing time spent on interpretation. Another limitation is the sample used. As it is not always possible to recruit sufficient numbers of highly trained individuals, such as consultant cardiologists due to various constraints, the sample selected for this study necessitated using individuals from a wide variety of clinical backgrounds, this conceivably has some impact on their approach to interpretation based on their differing training and professional backgrounds. Finally, the different group sizes generated when comparing accuracy (as we cannot know in advance who will make a correct or incorrect interpretation per ECG) makes direct comparisons challenging and such results should be interpreted with some caution.

## Conclusions

These approaches represent a different perspective, where computational techniques can be leveraged to support human interpretation and enhance human expertise, allowing the human to make the final decision. As such it appears that the examination of human expertise as a means to improve interpretation holds some promise in enhancing performance. As ECG interpretation is both a cognitive and visual process, the use of eye-tracking was examined as a possible methodology to extract such expertise from human interpreters. Findings indicate that clinical history in the form of history of presenting complaint does impact on accuracy and visual transitions in some cases at lead level. In contrast history has a greater cognitive and perceptual impact at the level of the waveform, changing how people react to the ECG presented. When considering accuracy, we again see greater differences in transition behavior at the level of the waveform, than the lead. Future analysis of eye-movements relating to ECG analysis should consider within lead visual behavior at the level of the waveform components.

## Supplementary information

Appendix

## Data Availability

Data and analysis code available from: https://github.com/IAM-lab/clinical-history.
