# Supplementary material for: Investigating the effect of clinical history before electrocardiogram interpretation on the visual behavior and interpretation accuracy of clinicians
Source: Sci Rep. 2019 Aug 5;9:11300. doi: 10.1038/s41598-019-47830-0 (PMC6683299; doi:10.1038/s41598-019-47830-0)
Supplement: Supplementary file 1 — Appendix [file 41598_2019_47830_MOESM1_ESM.docx]

**Investigating the effect of clinical history before electrocardiogram interpretation on the visual behavior and interpretation accuracy of clinicians**

Alan Davies^1,*^, Simon Harper^1^, Markel Vigo^1^ and Caroline Jay^1^

^1^School of Computer Science, University of Manchester, UK

**Corresponding author:**

Alan Davies

Office LF1,

Kilburn Building,

University of Manchester,

Manchester,

M13 9PL,

UK

**Email**: [alan.davies-2@manchester.ac.uk](mailto:alan.davies-2@manchester.ac.uk)

**Phone**: +44 (161) 275 7821

Simon Harper

Office 2.60,

Kilburn Building,

University of Manchester,

Manchester,

M13 9PL,

UK

Markel Vigo

Office 2.32,

Kilburn Building,

University of Manchester,

Manchester,

M13 9PL,

UK

Caroline Jay

Office 2.30,

Kilburn Building,

University of Manchester,

Manchester,

M13 9PL,

UK

**Keywords**: Eye-tracking; Electrocardiograms; ECG; EKG; Clinical history; visual behavior

**Word count**: 5629

| Appendix. Results of sub-group analysis for accuracy for both analysis methods | | | | | | |
| --- | --- | --- | --- | --- | --- | --- |
|  |  |  | **Top-down AOI** | | **Bottom-up AOI** | |
| **ECG** | **Presentation**  **groups** | **Sub-groups** | ***Hd*** | ***p-value*** | ***Hd*** | ***p-value*** |
| LBBB | last/last | correct/incorrect | 0.690 | 0.214 | 0.782 | 0.291 |
|  | first/first | correct/incorrect | 0.637 | 0.723 | 0.714 | 0.565 |
|  | first/last | correct/incorrect | 0.619 | 0.449 | 0.803 | <0.001** |
|  | first/last | incorrect/incorrect | 0.699 | 0.445 | 0.703 | <0.001** |
|  | first/last | correct/correct | 0.545 | 0.646 | 0.853 | <0.001** |
| Lateral STEMI | last/last | correct/incorrect | 0.543 | 0.860 | 0.825 | 0.704 |
|  | first/first | correct/incorrect | 0.512 | 0.976 | 0.767 | 0.581 |
|  | first/last | correct/incorrect | 0.438 | 0.993 | 0.868 | <0.001** |
|  | first/last | incorrect/incorrect | 0.530 | 0.980 | 0.847 | <0.001** |
|  | first/last | correct/correct | 0.556 | 0.900 | 0.845 | <0.001** |
| AF | last/last | correct/incorrect | 0.612 | 0.887 | 0.494 | 0.705 |
|  | first/first | correct/incorrect | 0.561 | 0.556 | 0.463 | 0.526 |
|  | first/last | correct/incorrect | 0.645 | 0.149 | 0.579 | <0.001** |
|  | first/last | incorrect/incorrect | 0.699 | 0.773 | 0.523 | <0.001** |
|  | first/last | correct/correct | 0.564 | 0.540 | 0.648 | <0.001** |
| RBBB | last/last | correct/incorrect | 0.645 | 0.529 | 0.764 | 0.876 |
|  | first/first | correct/incorrect | 0.617 | 0.435 | 0.748 | 0.247 |
|  | first/last | correct/incorrect | 0.660 | 0.186 | 0.797 | <0.001** |
|  | first/last | incorrect/incorrect | 0.646 | 0.698 | 0.776 | <0.001** |
|  | first/last | correct/correct | 0.671 | 0.065 | 0.806 | <0.001** |
| Inferior STEMI & AF | last/last | correct/incorrect | 0.569 | 0.941 | 0.874 | 0.543 |
|  | first/first | correct/incorrect | 0.563 | 0.436 | 0.816 | 0.862 |
|  | first/last | correct/incorrect | 0.615 | 0.337 | 0.806 | <0.001** |
|  | first/last | incorrect/incorrect | 0.596 | 0.775 | 0.856 | <0.001** |
|  | first/last | correct/correct | 0.565 | 0.360 | 0.888 | <0.001** |
| Anterior STEMI | last/last | correct/incorrect | 0.519 | 0.443 | 0.835 | 0.017* |
|  | first/first | correct/incorrect | 0.509 | 0.812 | 0.766 | 0.205 |
|  | first/last | correct/incorrect | 0.532 | 0.400 | 0.882 | <0.001** |
|  | first/last | incorrect/incorrect | 0.554 | 0.706 | 0.845 | <0.001** |
|  | first/last | correct/correct | 0.442 | 0.909 | 0.892 | <0.001** |
| High lateral STEMI | last/last | correct/incorrect | 0.616 | 0.612 | 0.523 | 0.041* |
|  | first/first | correct/incorrect | 0.636 | 0.703 | 0.489 | 0.390 |
|  | first/last | correct/incorrect | 0.743 | 0.564 | 0.578 | <0.001** |
|  | first/last | incorrect/incorrect | 0.616 | 0.393 | 0.622 | <0.001** |
|  | first/last | correct/correct | 0.656 | 0.314 | 0.592 | <0.001** |
| Inferolateral STEMI | last/last | correct/incorrect | 0.798 | <0.001** | 0.729 | 0.978 |
|  | first/first | correct/incorrect | 0.601 | 0.816 | 0.791 | 0.145 |
|  | first/last | correct/incorrect | 0.761 | 0.106 | 0.640 | 0.706 |
|  | first/last | incorrect/incorrect | 0.806 | 0.028* | 0.627 | 0.206 |
|  | first/last | correct/correct | 0.543 | 0.334 | 0.882 | <0.001** |
| Anterolateral STEMI | last/last | correct/incorrect | 0.650 | 0.455 | 0.685 | 0.94 |
|  | first/first | correct/incorrect | 0.562 | 0.767 | 0.744 | 0.602 |
|  | first/last | correct/incorrect | 0.674 | 0.346 | 0.693 | 0.826 |
|  | first/last | incorrect/incorrect | 0.523 | 0.403 | 0.525 | 1 |
|  | first/last | correct/correct | 0.474 | 0.687 | 0.833 | <0.001** |
| ***Note****: Hd = Hellinger distance, * = p < 0.05, ** = p < 0.001* | | | | | | |
